# Molecular diagnosis of trypanosomatids in *Didelphis marsupialis* reveals risk areas for *Trypanosoma cruzi* transmission and sympatric circulation with *T. rangeli* in the metropolitan area of Bucaramanga, Santander, Colombia

**DOI:** 10.1016/j.ijppaw.2025.101072

**Published:** 2025-04-14

**Authors:** Jeiczon Elim Jaimes-Dueñez, Vladimir Quintero-Sánchez, Andrea Ardila-Gélvez, Luz H. Patiño, Carlos M. Ospina, Ángela Patricia Jiménez-Leaño, Ian Sebastián Murcia-Cueto, Juan David Ramírez

**Affiliations:** a Grupo de Investigación en Ciencias Animales - GRICA, Facultad de Medicina Veterinaria y Zootecnia, Universidad Cooperativa de Colombia (UCC), Bucaramanga, Colombia; b Centro de Investigaciones en Microbiología y Biotecnología-UR (CIMBIUR), Facultad de Ciencias Naturales, Universidad del Rosario, Bogotá, Colombia; c Molecular Microbiology Laboratory, Department of Pathology, Molecular and Cell-Based Medicine, Icahn School of Medicine at Mount Sinai, New York City, NY, USA

**Keywords:** Chagas diseases, Leishmaniasis, Next-generation sequencing, Coinfection, Reservoir

## Abstract

The adaptation of wild animals to urban environments can lead to increased contact with humans and a higher risk of exposure to zoonotic agents. *Didelphis marsupialis* (common opossum) is an important reservoir of *Trypanosoma cruzi* and *Leishmania* spp., which commonly affect human populations in Latin America. Therefore, this study aimed to assess the frequency of trypanosomatid infections and characterize *T. cruzi* DTUs in common opossums from the Metropolitan Area of Bucaramanga (MAB), Santander, Colombia. A total of 70 individuals from four municipalities (Bucaramanga, Floridablanca, Girón, and Piedecuesta) were analyzed by PCR using blood samples, of which 14.3 % (95 % CI: 7.95–24.3 %) tested positive for trypanosomatids. Next-generation sequencing of 18S and Hsp70 genes in positive samples identified *T. cruzi* DTU TcI and *T. rangeli* in nine (12.9 %, 95 % CI: 6.91–22.66 %) and two (2.86 %, 95 % CI: 0.79–9.83 %) samples, respectively, including one case of co-infection (1.43 %, 95 % CI: 0.04–7.7 %). A heatmap revealed a high concentration of *T. cruzi*-positive cases in peripheral neighborhoods of Bucaramanga adjacent to forested areas. This study confirms the presence of an enzootic transmission cycle of *T. cruzi* in the MAB, highlighting the role of *D. marsupialis* as an important reservoir, particularly in peripheral neighborhoods of Bucaramanga. The sympatric circulation of *T. cruzi* and *T. rangeli* in opossums from the MAB introduces new epidemiological challenges for Chagas disease control in these areas, emphasizing the need for improved diagnostic strategies to differentiate both parasites in patients and epidemiological studies including vectors and reservoirs.

## Highlights

• Molecular analyses in opossums from MAB reveal a lower *T. cruzi* infection rate.
• The study confirms an enzootic *T. cruzi* cycle in MAB, especially in peripheral neighborhoods.
• Analyses reveal the sympatric circulation of *T. cruzi* and *T. rangeli* in opossums from MAB.

## Graphical abstract

**Figure:**
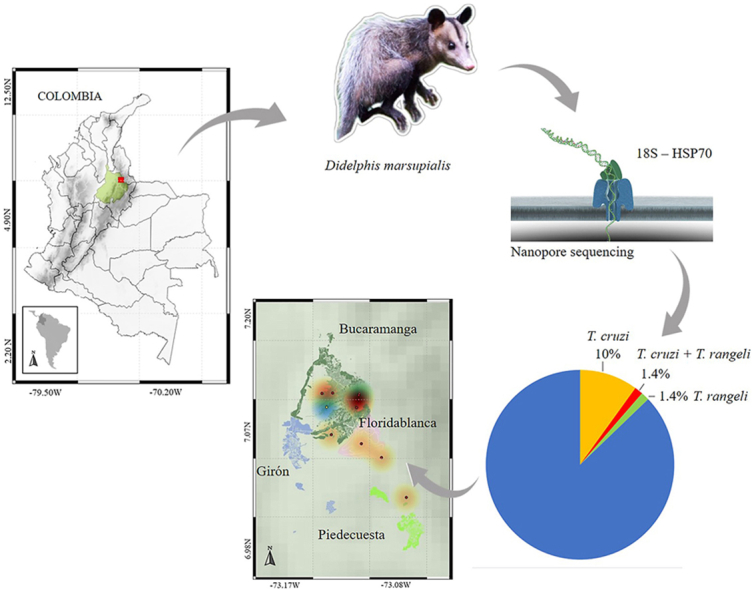

## 1 Introduction

Trypanosomatids are protozoan parasites of major medical and veterinary importance. They cause serious diseases in humans such as sleeping sickness (*Trypanosoma brucei*), Chagas disease (CD) (*Trypanosoma cruzi*) and Leishmaniasis (*Leishmania* spp.) (Kaufer et al., 2017). In the Americas, CD and leishmaniasis are particularly notable due to their high incidence, primarily affecting developing communities and countries with significant migrant populations from rural areas (Avaria et al., 2022; Cosma et al., 2024). According to the World Health Organization, an estimated eight million people in Latin America are affected by CD, leading to approximately 12,000 deaths every year (WHO, 2024). In recent decades, shifting migration patterns have altered the global distribution of CD, contributing to an increase in cases in regions such as Europe, North America, and the Western Pacific, making it a growing global health problem (Gonzalez-Sanz et al., 2023; Pinto Dias, 2013).

In endemic areas, transmission of *T. cruzi* primarily occurs through contact with the feces of infected triatomine bugs during blood meal (Hemiptera: Reduviidae) (Cantillo-Barraza et al., 2025). Secondary transmission routes include the consumption of contaminated food or beverages, vertical transmission, blood transfusions, organ transplantation, and accidental exposure in laboratory settings (Beatty et al., 2024; Christian et al., 2025). In Colombia, significant progress has been made in controlling the disease through the implementation of a vector-borne transmission interruption plan in hyperendemic areas. However, the persistent circulation of *T. cruzi* in reservoirs within urban environments poses an ongoing transmission risk.

Wild mammals of the genus *Didelphis* (opossums), particularly *D. marsupialis*, *D. albiventris* and *D. virginiana*, are key reservoirs for trypanosomatids of the genera *Leishmania* and *Trypanosoma*, serving as primary hosts of *T. cruzi* in the Americas (Cantillo-Barraza et al., 2015; Acosta et al., 2017; Zecca et al., 2020), particularly for the TcI Discrete Typing Unit (DTU) (Zingales et al., 2012; Calvopina et al., 2020). Due to their synanthropic and generalist behavior, along with continuous exposure to vectors, *D. marsupialis* populations contribute to maintaining endemic transmission cycles in domestic areas of Central and South America and may facilitate *T. cruzi* dissemination to humans through scent gland secretions (Cantillo-Barraza et al., 2024). In Colombia, studies have reported urban CD outbreaks linked to contamination with opossum secretions in areas free of insect transmission (Cantillo-Barraza et al., 2020; Gutiérrez et al., 2024), posing new challenges for the control of CD.

Although *T. cruzi* is the primary trypanosomatid infecting *D. marsupialis*, *T. rangeli* also infects the same vertebrate hosts and vectors, resulting in overlapping transmission cycles (Gottdenker et al., 2016; Vallejo et al., 2015). In some areas of Latin America and the Caribbean, human infection with *T. rangeli* is more frequent than *T. cruzi*, and mixed infections are commonly reported (Saldaña et al., 2005; Vergara-Meza et al., 2022). Despite being considered non-pathogenic to humans, *T. rangeli* is epidemiologically significant, as it can cause false-positive results in microscopic and serological tests used for *T. cruzi* diagnosis (de Moraes et al., 2008), given that both parasites share approximately 60 % of their soluble antigenic composition (Saldaña and Sousa, 1996). Therefore, research on *T. rangeli* and its differentiation from *T. cruzi* is crucial for improving the epidemiological understanding of CD in endemic areas.

The Metropolitan Area of Bucaramanga (MAB), Santander, is an endemic region for CD and has been the focus of outbreaks of both CD and visceral leishmaniasis, primarily linked to vector-borne transmission. The main vectors reported for CD transmission in the MAB include intrusive populations of *Panstrongylus geniculatus* and *Rhodnius pallescens* (Reyes et al., 2017), while *Lutzomyia ovallesi* and *Lutzomyia gomezi* have been suggested as potential vectors of *Leishmania* spp. (Sandoval et al., 1998). Although epidemiological studies suggest that *D. marsupialis* plays a key role in CD transmission in this area (Reyes et al., 2017), contemporary data on the frequency and genotypic diversity of zoonotic trypanosomatid infections in *D. marsupialis* in MAB remain scarce. Accordingly, this study aims to assess the frequency of trypanosomatid infections and to characterize *T. cruzi* DTUs in common opossums from the MAB.

## 2 Material and methods

### 2.1 Ethics statement

This research was conducted in accordance with the Colombian code of practice for the care and use of animals for scientific purposes, as outlined in Law 84 of 1989 and Law 576 of 2000. The study was considered “Research with Minimal Risk”. It was endorsed by the Ethics Committee of the Cooperative University of Colombia approved the study, with concept INV3553 in the Act No. 001 of 2021. The Regional Autonomous Corporation of the Metropolitan Area of Bucaramanga (CDMB) acted in accordance with the government regulations established in Decree 393 of 1991, for scientific and technological activities.

### 2.2 Study area and sample size

**Fig. 1:**
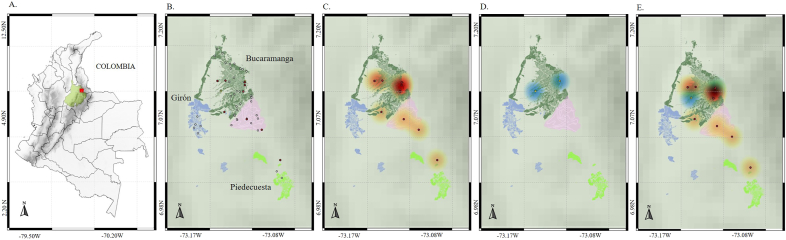
Heatmaps of trypanosomatid infections in *Didelphis marsupialis* from the Metropolitan Area of Bucaramanga, Santander. (A) Map of Colombia showing the Santander department (green) and Metropolitan Area of Bucaramanga (red). (B) Geographic locations where specimens of *Didelphis marsupialis* were rescued. Gray points represent individuals negative for infection, while red and yellow points indicate individuals positive for *T. cruzi* and *T. rangeli*, respectively. (C) Geographic distribution of individuals positive for *T. cruzi*. (D) Geographic distribution of individuals positive for *T. rangeli*. (E) Overlay of individuals positive for *T. cruzi* and *T. rangeli*.

The MAB is the fifth urban metropolis from Colombia composed by the cities of Bucaramanga, Floridablanca, Girón, and Piedecuesta with a population of approximately 1,341,694 inhabitants (Fig. 1A and B). The vegetation is a transition between the Tropical Dry Forest and Rainforest Premontane, with an altitude ranging from 706 to 959 m and an annual precipitation of 4426 mm. The climate is categorized as Tropical Rainforest (Af according to Köppen climate classification) (IDEAM, 2022).

The study population consisted in specimens of *D. marsupialis* attended by the Wildlife Care Center (CAV) of the CDMB between April 2023 and April 2024. These animals are brought to the CAV following community reports regarding their presence in homes, workplaces, or public spaces. Additionally, some specimens are transferred to the center by authorities and rescue teams during seizures or rescue operations. The sample size was calculated based on records of *D. marsupialis* specimens attended by the CAV in 2021 and 2022 (250 individuals), an expected infection rate of 48 % as reported by Rodríguez-Monguí et al. (2019), a 95 % confidence level, and a 5 % margin of error. A total sample size of 77 individuals was determined for the study. Exclusion criteria included neonatal animals (weighing less than 55 g), pregnant and lactating females, and animals in fragile clinical conditions where the sampling process could endanger their survival.

### 2.3 Sampling process

Individuals were anesthetized via inhalation with isoflurane, using an initial dose of 4 % and a maintenance dose of 0.5–1 %. After anesthesia, weight was measured using a digital scale with a 20 kg capacity (Vibra, Terrace, USA). Specimens were classified by weight as neonates (less than 55 g), juveniles (55–450 g), and adults (over 450 g) (Flórez-Oliveros and Vivas-Serna, 2020). To assess health status, heart rate, respiratory rate, temperature, and mucosal color were measured, and palpation of the musculoskeletal system was performed to check for injuries. Clinical condition was classified as healthy (apparently healthy animal), compromised (animal with moderate or minor injuries that can be managed), or delicate (critically ill animal with severe injuries or fractures that are life-threatening) (Flórez-Oliveros and Vivas-Serna, 2020).

A blood sample of 0.25 mL was collected from the coccygeal vein of each animal using a 23G x 1″ needle, transferred into 0.5 mL EDTA tubes, and stored at −20 °C until further processing. Genomic DNA was extracted from 200 μL of blood using the Genomic DNA Purification Kit (CorpoGen, Bogotá, Colombia) following the manufacturer's protocol. The extracted DNA was diluted with 100 μL of elution buffer and stored at −20 °C until molecular analysis. DNA quantity and quality were assessed using a NanoDrop 2000 spectrophotometer (Thermo Fisher Scientific, Massachusetts, USA).

To avoid the potential resampling of specimens, a marking system was implemented using an injectable microchip according the protocol ISO 11784 (EU, 2025).This marking procedure was carried out in accordance with Resolution 1172 of 2004 of Colombian government, which establishes the National System for the Identification and Registration of Wildlife Specimens in *Ex Situ* Conditions. After that, individuals were reintegrated into peripheral forests of the MAB according to CAV protocols.

### 2.4 Molecular analysis

Two conventional PCR assays were conducted to detect trypanosomatid infections. The first targeted the microsatellite region of *T. cruzi* using previously described primers TZ1 and TZ2 (Ramírez et al., 2015; Sturm et al., 1989), while the second targeted the Hsp70 gene, a marker commonly used to detect and identify *Leishmania* species, employing previously described primers HSP70F and HSP70R (Hernández et al., 2014). Each PCR reaction was performed in a 25-μL volume containing 1X reaction buffer (100 mM Tris-HCl, 50 mM KCl, pH 8.8), 0.2 mM dNTPs, 1.5 mM MgCl2, 0.4 μM of each primer, 0.625 U of Taq polymerase (CorpoGen, Bogotá, Colombia), and 2 μL (20 ng) of DNA template. The PCR products were resolved on a 2 % agarose gel, stained with EZ-Vision (AMRESCO, Solon, USA), and visualized under ultraviolet light. Samples were considered positive for *T. cruzi* or *Leishmania* spp. when bands of 188 bp or 337 bp were observed, respectively. The positive controls consist of DNA extracted from the blood samples of *D. marsupialis* naturally infected with *T. cruzi* and *L. infantum*, respectively. Negative controls were derived from DNA samples of an opossum previously characterized as negative for trypanosomatids.

### 2.5 Next-generation sequencing

For each sample positive for trypanosomatids, a ∼900 bp fragment of the first section of the 18S rRNA gene and 337 bp of the Hsp70 gene were amplified using conventional PCR, following the primers and protocols described by (Patiño et al., 2023). The amplification products were validated through agarose gel electrophoresis. After confirmation, the amplicons were sequenced using Oxford Nanopore technology on the MinION platform with R10.4.1 flow cells. Base calling was performed using the “Super Accuracy” mode in the Dorado software v.0.6.0 (https://github.com/nanoporetech/dorado?tab=readme-ov-file). The resulting FASTQ files were assessed for quality and subjected to taxonomic assignment using the bioinformatics pipeline proposed by (Cruz-Saavedra et al., 2024). The data were filtered based on specific parameters: for the 18S rRNA gene, sequences with a “Hitlength” ≥ 500 bp and a “Querylength” between 800 and 1100 bp were retained. For the Hsp70 gene, sequences were filtered using a “Hitlength” ≥ 200 bp and a “Querylength” between 200 and 500 bp.

### 2.6 Data analyses

The frequency of infection was estimated with a 95 % confidence interval (CI) according to Brown et al. (2001). To assess the epidemiological variables associated with infection for each species, the molecular diagnosis results were analyzed through bivariate analysis (χ^2^ test), considering five independent variables: sex, age, clinical conditions, geographical origin, and socioeconomic status. The last two variables correspond to the geographic and socioeconomic characteristics of the area where each specimen was collected. Socioeconomic status was determined based on the official stratum divisions as follows: level 1, lower-low; level 2, low; level 3, upper-low; level 4, medium; level 5, medium-high; and level 6, high (Farnsworth, 2017). All statistical analyses were conducted using SPSS software v.18.0. The spatial distribution of cases within the MAB was represented through heatmaps created using the heatmap tool in QGIS version 3.4.0.

## 3 Results

### 3.1 Description of sample and frequency of infection

During the study period, blood samples were collected from 70 *D. marsupialis* specimens. Among them, 61.4 % derived from Bucaramanga, 27.1 % from Floridablanca, 7.1 % from Girón, and 4.2 % from Piedecuesta. Clinical examinations revealed that 50 % were in good health, 22.8 % were compromised, and 27.1 % had delicate clinical condition. In terms of age, 67.1 % were classified as adults, while the remaining were juveniles. The sex distribution was evenly split at 50 %.

A total of 10 samples (14.3 %, 95 % CI = 7.95–24.3 %) tested positive for trypanosomatids. Of these, nine were positive for the Hsp70 marker and eight for the *T. cruzi* nuclear satellite marker. Seven samples tested positive for both markers. Next-generation sequencing of the 18S fragment identified nine positive samples: nine were positive for *T. cruzi* DTU TcI (12.9 %, 95 % CI: 6.91–22.66 %), two for *T. rangeli* (2.86 %, 95 % CI: 0.79–9.83 %), and one showed a coinfection (1.43 %, 95 % CI: 0.04–7.7 %). Similar results were obtained from Hsp70 sequencing, which detected *T. cruzi* DTU TcI in eight samples (11.4 %, 95 % CI: 3.98–18.88 %) and one case of coinfection with *T. rangeli*. Eight of the ten positive animals have compromised or delicate clinical condition (Table 1). No infections with *Leishmania* spp. were detected.

**Table 1:** Taxonomic classification of 18S rRNA and Hsp70 reads obtained from *Didelphis marsupialis* infected with *Trypanosoma* spp., in the Metropolitan Area of Bucaramanga, Santander, between April 2023 and April 2024.

ID	Sex	Clinical condition	Origin	18S			Hsp70		
				Number of reads assigned	Percent of reads assgined	Taxon assigned	Number of reads assigned	Percent of reads assgined	Taxon assigned
9	Female	Healthy	Bucaramanga	823	99 %	*T. cruzi* (TcI)	2068	99 %	*T. cruzi*
10	Male	Delicate	Bucaramanga	–	–	–	528	100 %	*T. cruzi*
12	Female	Delicate	Bucaramanga	2889	99 %	*T. cruzi* (TcI)	7772	100 %	*T. cruzi*
13	Male	Delicate	Floridablanca	1145	97 %	*T. cruzi* (TcI)	10648	99 %	*T. cruzi*
51	Male	Compromised	Piedecuesta	9607	98 %	*T. cruzi* (TcI)	–	–	–
53	Female	Healthy	Bucaramanga	5886/14150	29 %/70 %	*T. cruzi* (TcI)/*T. rangeli*	96/90	51 %/49 %	*T. cruzi/T. rangeli*
55	Male	Delicate	Floridablanca	1375	98 %	*T. cruzi* (TcI)	34086	99 %	*T. cruzi*
58	Female	Delicate	Bucaramanga	13083	99 %	*T. cruzi* (TcI)	4094	99 %	*T. cruzi*
63	Female	Compromised	Bucaramanga	117	99 %	*T. rangeli*	–	–	–
65	Female	Compromised	Bucaramanga	823	99 %	*T. cruzi* (TcI)	1769	97 %	*T. cruzi*

### 3.2 Epidemiological variables associated to the infection

Bivariate analyses revealed a statistically significant association between *T. cruzi* infection with the age of the specimens and socioeconomic status of the area where they were rescued, with a higher frequency of infection observed in adults and individuals rescued from areas with a high socioeconomic status (level 4). *T. rangeli* infection showed no significant association with any independent variable (Table 2). The geographic distribution of *T. cruzi* infection revealed positive cases in all municipalities except Girón (Fig. 1C–E), with heatmap analyses indicating a high concentration of positivity in the southeastern region of Bucaramanga, specifically in peripheral neighborhoods adjacent to forest areas (Fig. 1C–E). A similar pattern was observed for *T. rangeli* infection, where positive cases were exclusively reported in Bucaramanga (Fig. 1D).

**Table 2:** Bivariate analyses of infection by *Trypanosoma* spp., in *Didelphis marsupialis* from the Metropolitan Area of Bucaramanga, Santander, between April 2023 and April 2024.

Variable	n	*T. cruzi* (+)	Frequeny	χ2 test	p- value	*T. rangeli* (+)	Frequeny	χ2 test	p- value
Sex									
Female	35	5	14.2 %	0.127	0.721	2	5.7 %	2.059	0.151
Male	35	4	11.4 %			0	0 %		
Ege									
Young	23	0	0 %	5.054	0.024∗	0	0 %	1.008	0.315
Adult	47	9	19.1 %			2	4.2 %		
Clinical condition									
Healthy	35	2	5.7 %	4.667	0.096	1	2.8 %	1.222	0.542
Compromised	16	2	12.5 %			1	6.2 %		
Delicate	19	5	26.3 %			0	0 %		
Origin									
Bucaramanga	44	6	13.6 %	1.933	0.586	2	4.5 %	7.208	0.065
Floridablanca	18	2	11.1 %			0	0 %		
Girón	5	0	0 %			0	0 %		
Piedecuesta	3	1	33.3 %			0	0 %		
Socioeconomic status									
Level 3	26	3	19.2 %	6.938	0.031∗	1	3.8 %	1.991	0.369
Level 4	10	2	10 %			0	0 %		
Level 5	7	4	42.8 %			1	14.2 %		

## 4 Discussion

Human activities such as urbanization, deforestation, wildlife exploitation, and tourism, along with global climate changes, not only alter natural landscapes but also serve as driving forces for the zoonotic spillover (Esposito et al., 2023). The establishment of enzootic cycles near human populations has been linked to a rise in zoonotic infections as those caused by trypanosomatids (Cantillo-Barraza et al., 2022). This study confirms the presence of an enzootic transmission cycle of *T. cruzi* in the MAB and highlights the role of *D. marsupialis* as a reservoir of the parasite in these urban areas, posing a significant risk of transmission to human populations. Additionally, we provided evidence of the sympatric circulation of *T. cruzi* and *T. rangeli* in Bucaramanga.

Regarding the frequency of *T. cruzi* infection, the results obtained here were higher than those recently reported for wild mammal species in various regions of Colombia, where a meta-analysis suggests an average infection rate of 6.94 % (Rodríguez-Monguí et al., 2019). Similarly, the results are slightly higher than those reported for *D. marsupialis* in urban areas of Manaus, Brazil (12.4 %), where human intervention and habitat fragmentation reduce contact with the parasite's vectors (Magalhães et al., 2021). In contrast, our results are lower than those observed in rural populations of *D. marsupialis* in Los Montes de María and Margarita Island in the Colombian Caribbean, where infection rates ranged from 30 % to 61.5 % (Ardila et al., 2023). Although invasive triatomines such as *Panstrongylus geniculatus* and *Rhodnius pallescens* are frequently found in the MAB with high *T. cruzi* infection rates (>50 %), likely originating from periurban forests where the sylvatic cycle is maintained (Reyes et al., 2017), the lower infection frequency in *D. marsupialis* from these areas compared to rural populations could be explained by reduced exposure to infected triatomines. This reduction could result from the greater availability of alternative food sources in urban areas, such as food waste, fruits, carrion and others, which might lower reliance on infected vectors. However, the frequent isolation of triatomine and opossum populations due to habitat fragmentation, as observed in urban habitats from Brazil (Magalhães et al., 2021), could also explain this pattern.

The MAB is considered an endemic area for CD, with 19 cases reported in 2023 (Sivigila, 2023), including sporadic oral outbreaks caused by TcI and associated with contaminated food containing triatomine feces or opossum excrement (Hernández et al., 2009; Velásquez-Ortiz and Ramírez, 2020). Although opossum excrement was not analyzed in this study, our findings suggest that *D. marsupialis* plays a key role as a reservoir of TcI in domestic areas, sustaining an enzootic cycle in these cities. This cycle appears to be more intense in peripheral neighborhoods of Bucaramanga, which are adjacent to forest where the sylvatic cycle of *T. cruzi* persists (Reyes et al., 2017), suggesting that *D. marsupialis* becomes infected in these areas and introduces the parasite in peripherical areas of the city due to its anthropophilic behavior. Notably, 70 % of Chagas disease cases reported in 2023 occurred in Bucaramanga, highlighting the need to establish surveillance systems in periurban neighborhoods and implement educational strategies to reduce human-opossum contact in forest parks, schools, and green areas, that reduce the risk of transmission. Nevertheless, further spatiotemporal analyses in both humans and animals are necessary to better understand the transmission dynamics in these urban settings.

Human infections with *T. rangeli* are common in Latin America, and the parasite is considered non-pathogenic to humans (Vallejo et al., 2015). This species has been reported in specific departments at the Caribbean and Andean regions, infecting the same hosts and vectors as *T. cruzi* (Ardila et al., 2022; Vallejo et al., 2015). The first report of *T. rangeli* in the MAB introduces a new challenge for CD control, underscoring the need for specific diagnostic methods in humans and the importance of studying the transmission dynamics of this parasite into these cities. Although co-infection with *T. cruzi* and *T. rangeli* is frequently detected in triatomine vectors (Gottdenker et al., 2016; Wu et al., 2020), it is relatively rare in mammals (Dario et al., 2022), with limited case reports available. The detection of an opossum co-infected with these parasites raises critical questions about the genotypes capable of persisting in co-infection scenarios in mammals. This finding could complicate the epidemiology of CD in the MAB, as co-infection with both parasites may influence the progression of CD, such as observed in recent oral outbreaks in Acre, Brazil (Vergara-Meza et al., 2022).

Finally, some studies have shown that *T. cruzi* infection may present a health risk to opossums, leading to effects such as lymphocytosis, eosinophilia, and cyst formation in various tissues, including the heart and skeletal muscles (Arruda et al., 2019). These pathological changes can negatively impact the body condition of infected animals (Zecca et al., 2020). In this study, although the clinical status of the animals was associated with the number and severity of injuries, such as wounds and fractures, 80 % of *T. cruzi*-positive individuals exhibited a compromised or delicate clinical condition. These findings suggest that stress related to traumatic events could increase parasitemia in chronically infected animals, potentially heightening the risk of transmission to humans. Therefore, special biosecurity measures should be implemented when handling this group of animals in rescue centers, especially those with poor clinical conditions. Further clinical analyses are necessary to validate this hypothesis.

In conclusion, this study confirms the presence of an enzootic transmission cycle of *T. cruzi* in the MAB, underscoring the role of *D. marsupialis* as an important reservoir in urban environments. The findings indicate a higher frequency of *T. cruzi* infection in peripheral neighborhoods of Bucaramanga adjacent to forested areas, where the sylvatic cycle of the parasite likely persists. The sympatric circulation of *T. cruzi* and *T. rangeli* in opossums from MAB, including cases of co-infection, introduces new epidemiological challenges for CD control in these cities, particularly for diagnostic centers and surveillance systems.

## CRediT authorship contribution statement

**Jeiczon Elim Jaimes-Dueñez:** Formal analysis, Data curation, Conceptualization, Jeiczon Jaimes, Methodology, Investigation, Funding acquisition, Data curation, Conceptualization. **Vladimir Quintero-Sánchez:** Investigation, Funding acquisition, Formal analysis. **Andrea Ardila-Gélvez:** Methodology, Investigation, Funding acquisition. **Luz H. Patiño:** Methodology, Investigation. **Carlos M. Ospina:** Investigation, Funding acquisition. **Ángela Patricia Jiménez-Leaño:** Supervision, Project administration, Investigation, Funding acquisition. **Ian Sebastián Murcia-Cueto:** Methodology, Investigation. **Juan David Ramírez:** Project administration, Methodology, Investigation, Funding acquisition, Formal analysis, Data curation.

## Data availability

Data will be made available on request at jeiczon.jaimes@campusucc.edu.co.

## Declaration of competing interest

The author is an Editorial Board Member/Editor-in-Chief/Associate Editor/Guest Editor for *[International Journal for Parasitology: Parasites and Wildlife]* and was not involved in the editorial review or the decision to publish this article.

The authors declare the following financial interests/personal relationships which may be considered as potential competing interests: Jeiczon Elim Jaimes-Dueñez, Vladimir Quintero-Sánchez, Andrea Ardila-Gélvez, Luz H. Patiño, Carlos M Ospina, Ángela Patricia Jiménez-Leaño, Ian Sebastián Murcia-Cueto, Juan David Ramírez.
